# The group home as moral laboratory: tracing the ethic of autonomy in Dutch intellectual disability care

**DOI:** 10.1007/s11019-020-09991-y

**Published:** 2021-01-04

**Authors:** Simon van der Weele, Femmianne Bredewold, Carlo Leget, Evelien Tonkens

**Affiliations:** 1 Department Citizenship and Humanisation of the Public Sector, University of Humanistic Studies, Kromme Nieuwegracht 29, 3512 HD Utrecht, The Netherlands; 2 Department Care Ethics, University of Humanistic Studies, Utrecht, The Netherlands

**Keywords:** Moral laboratory, Care, Autonomy, Disability, Intellectual disability, Governmentality

## Abstract

This paper examines the prevalence of the ideal of “independence” in intellectual disability care in the Netherlands. It responds to a number of scholars who have interrogated this ideal through the lens of Michel Foucault’s vocabulary of governmentality. Such analyses hold that the goal of “becoming independent” subjects people with intellectual disabilities to various constraints and limitations that ensure their continued oppression. As a result, these authors contend, the commitment to the ideal of “independence” – the “ethic of autonomy” – actually threatens to become an obstacle to flourishing in the group home. This paper offers an alternative analysis. It does so by drawing on a case study taken from an ethnographic study on group home life in the Netherlands. Briefly put, the disagreement stems from differing conceptualizations of moral life. Put in the vocabulary of moral anthropologist Cheryl Mattingly, the authors propose to approach the group home more from a “first-person” perspective rather than chiefly from a “third-person” perspective. They then draw on Mattingly to cast the group home as a “moral laboratory” in which the ethic of autonomy is not just reproduced but also enacted, and in which the terms of (in)dependence constantly get renegotiated in practice. What emerges is not only a new perspective on the workings of the “ethic of autonomy” in the group home, but also an argument about the possible limitations of the vocabulary of governmentality for analysing care practices.

## Prelude: three scenes from group home life

We would like to start this paper by offering a series of observations. Each of these took place in a group home for people with intellectual disabilities in the Netherlands during our ethnographic fieldwork in 2017 and 2018.

First, the following dinnertime scene from a group home.

Support worker Uma informs the residents that she has finished cooking dinner. The residents head to their individual apartments, to return moments later with a plate and cutlery. Resident Rowena explains that they eat their meals from their own plates, using their own cutlery. She turns to Uma for confirmation. Uma nods. “That’s part of being independent, isn’t it?”, she says. “Having your own things.”

Second, the following scene from a trip to the general store.

Support worker Jessica takes resident Erdem shopping for forks. She tells Erdem to pick and choose the forks he wants. Jessica is the first to find the isle with cutlery. She attempts to have Erdem choose. He picks. Jessica decides on a larger size. Then Jessica asks Erdem how many forks to buy. Erdem wants two. Jessica suggests buying four, for when visitors come to eat. Again, Erdem tells her he wants to buy two forks. Jessica briefly seems to hesitate, then decides to purchase four forks anyway.

Third, the following scene from a bedtime routine.

Support worker Eric watches resident Dennis brush his teeth. When Dennis is finished, we get ready to leave his room. As I’m walking away towards the hallway, Eric quietly beckons me to come back. Together we wait by the bathroom door. “I’m in the shower!”, Dennis calls out a moment later. Now Eric and I leave Dennis’s room. Eric explains that Dennis wants to shower by himself. To make sure he bathes, Dennis and the staff have agreed that a support worker can wait outside his bathroom until he’s entered the shower.

What ties these scenes together, in our view, is a certain preoccupation with the idea of “independence”. In all of them, its participants seem to espouse “independence” as a value to some degree: it resides in eating from your own plates and cutlery, choosing your own cutlery, and in taking a shower by yourself. Yet each scene also contains habits, behaviors, or tendencies seemingly at odds with “independence”. Uma cooks. Jessica decides. Eric supervises Dennis. An outsider might raise an eyebrow at the sight of some of the apparent contradictions in these scenes. This paper is an attempt to make sense of them. We return to these scenes in the conclusion.

## Introduction: “independence” in the group home

The idea (and ideal) of “independence” looms large in intellectual disability care in many countries in the Global North. It is invoked in ideals like autonomy and self-determination, which feature prominently in the UN Convention of the Rights of Persons with Disabilities, as well as in widely used quality of life measures for people with intellectual disabilities (Schalock 2004; Buntinx and Schalock 2010). It is also invoked in the contemporary jargon of many Western welfare states, which tend towards concepts like choice, responsibility, and self-reliance (Bredewold 2020; Newman 2011). While these vocabularies have different histories, they are bound by a promise of independence: from carers, for instance, or from the state.

This proliferation of the ideal of independence in intellectual disability care policies and practices is often understood as an effect of the deinstitutionalization movement, which, from the 1960s onwards, sought to close down large-scale care facilities in favour of living arrangements “in the community” (Pols et al. 2017). The narrative is often told as some version of the following: people with intellectual disabilities used to live their lives in dehumanizing conditions, in which their individuality was neither recognized nor developed. Moving out of the institution to live an “independent” life ended the paternalism associated with the institution and began to restore dignity and autonomy for people with intellectual disabilities. Henceforth, they would be able to engage in projects of self-realisation, with the benevolent support of trained care workers. While several philosophers have questioned the dominance of the ideal of “independence” in the care for people with intellectual disabilities at the expense of concepts such as dependency and relationality (Meininger 2001; Reinders 2002; Kittay 2011, 2019), some variation of this narrative often informs contemporary policy and practice (Meininger 2010). It also provides assumptions to a substantial amount of research in the social sciences (Altermark 2017).

However, this narrative has also faced scrutiny from another angle. A number of scholars of intellectual disability have interrogated the ideal of “independence” in intellectual disability care through the lens of Michel Foucault’s vocabulary of governmentality (Levinson 2005, 2010; Dowse 2009; Graham 2010; Drinkwater 2015; Altermark 2017, 2018; Munson 2020). Such analyses share the observation that life for people with intellectual disabilities is a constant project of “becoming independent”, as they are encouraged (or required) to adopt and internalize norms of autonomous personhood. Drawing on Nikolas Rose (1999), Jack Levinson (2010) speaks of an “ethic of autonomy” to refer to this imperative for independence. At the same time, however, since persons with intellectual disabilities are (in Levinson’s striking formulation) “thought of as free yet incapable of freedom” (2010, xiii), the goal of becoming independent will never be realized – it is an “ever-receding goal” (2010, 52). In this way, the ethic of autonomy that characterizes disability care does not deliver on its promise but does impose various constraints and limitations on people with intellectual disabilities to which they need to comply. In effect, these scholars suggest, while the disciplinary techniques of the total institution are (mostly) a thing of the past, the lives of people with intellectual disabilities continue to be the subject of control. These analyses echo James Trent’s famous observation that care and control are “interrelated and interdependent” factors in intellectual disability care (1994, 280). “Independence”, we contend, thus emerges as what Lauren Berlant (2011) would call “cruel optimism”, a concept that designates commitments to dreams or desires that seem vital for one’s flourishing but actually hinder it.

Similar to the works outlined above, this paper seeks to make sense of the prevalence of the ideal of “independence” in intellectual disability care in terms of the ethic of autonomy. It does so by drawing on ethnographic data from a research project on the significance of dependency in the lives of people with intellectual disabilities living in group homes in the Netherlands (Van der Weele 2020). However, while we think that the vocabulary of governmentality is highly instructive in making sense of the care practices we observed, we have reason to disagree with the cruel optimism thesis. Briefly put, this disagreement stems from how these authors conceptualize moral life. Put in the vocabulary of moral anthropologist Cheryl Mattingly (2014), we suggest they approach the group home too much from a “third-person” and not enough from a “first-person” perspective. As an alternative, we draw on Mattingly to cast the group home as a “moral laboratory” in which the ethic of autonomy is not just reproduced but also *enacted*, and in which the terms of (in)dependence constantly get renegotiated in practice. While the outcome of such adaptations may still be tragic, we contend, they do not necessarily amount to cruel optimism.

In what follows, we first turn to the arguments of Levinson and others, exploring the analysis of group home life in terms of an ethic of autonomy and how these lend credence to the cruel optimism thesis. Then we turn to our fieldwork. We will introduce a series of observations involving Jos, a resident of group home The Home, and Valerie, who is employed by The Home as support staff.^1^ We will analyse these scenes using the concept of the ethic of autonomy, first from a “third-person” and then from a “first-person” perspective. Afterwards, we draw on these analyses to probe the viability of the cruel optimism thesis and present our alternative – the moral laboratory thesis. In doing so, what will emerge is not only a new perspective on the workings of the ethic of autonomy in the group home, but also an argument about the possible limitations of the vocabulary of governmentality for analysing care practices.

## Governmentality and the ethic of autonomy

The concept of governmentality finds its origins in Foucault (e.g. Foucault 1982, 2009, 2010) and has been further elaborated by authors like Nikolas Rose (1999; 2005), Thomas Lemke (2002) and Mitchell Dean (2010). In what follows, we rely principally on Rose, as the concept of the ethic of autonomy is his. Foucault himself gave many definitions for “governmentality”, but a famous and widely-used one is the “conduct of conduct” (2014, 12).^2^ Rose characterizes governmentality as “all endeavours to shape, guide, direct the conduct of others… And it also embraces the ways in which one might be urged and educated to bridle one’s own passions, to control one’s own instincts, to govern oneself” (1999, 3). Governmentality thus refers to exercises of power that are geared towards (not inhibiting but) shaping the conduct of subjects in such a way that the direction of power and the life projects of subjects cohere, so that power is experienced as freedom. In this way, governmentality is an addendum to our vocabulary of the exercise of power in terms of domination. According to Rose, domination “is to ignore or to attempt to crush the capacity for action of the dominated”; governing, on the other hand, “is to recognize that capacity for action and to adjust oneself to it” (1999, 4). Governance, in other words, presupposes and acts upon the *freedom* of subjects, as opposed to denying or crushing it. This great irony – our condition of being “governed through our freedom” (Rose 1999, 62) – is a central paradox for the study of governmentality.

Authors like Jack Levinson (2005; 2010), Chris Drinkwater (2015), and Niklas Altermark (2017; 2018) use the conceptual framework of governmentality to look at the imbrication of power and care in contemporary group home life for people with intellectual disabilities. By doing so, their research is part of a broader attempt to understand intellectual disability as a matter of power and knowledge in Foucault’s sense (Yates 2015; Spivakovsky 2017; Munson 2020). While these writers each provide distinct analyses, the general argument is something like this: while deinstitutionalization (and the concomitant advent of the group home) has led to a decrease in the exercise of “traditional” coercive power in the lives of people with intellectual disabilities, the ideal of independence brings along new forms of governing that reinforce the coercion, restraint, and control to which they had previously been subjected.

Levinson links the embrace of the ideal of independence to what he calls an ethic of autonomy, a concept he borrows from Rose. In this ethic of autonomy, writes Levinson, “living well, living right, is defined by the attention we pay to ourselves … how we know and act on ourselves in terms of our own potential and individuality” (2010, xi). Rose argues that the ideal of freedom is foundational for our self-understanding as subjects and thus for the projects we are driven to pursue, both individually and collectively. In other words, since we think of ourselves as free, the pursuit of freedom and self-actualization becomes a social and cultural imperative. The ethic of autonomy refers to a set of norms that.

Operate a regime of the self where competent personhood is thought to depend upon the continual exercise of freedom, and where one is encouraged to understand one’s life, actually or potentially, not in terms of fate or social status, but in terms of one’s success or failure acquiring the skills and making the choices to actualize oneself. (1999, 87).

The subject, conceived of principally as a *free* subject, is to work on this freedom, develop it, in a continuous project of individualization and self-actualization. In this way, argues Rose – and this is again the central paradox – the ethic of autonomy *obliges us to be free*. And since this ethic assumes subjects to be free, autonomous agents, failing to exercise this autonomy properly is also a matter of personal responsibility. If we fail to be free in the right way, we have ourselves to blame.

For Levinson, the care for people with intellectual disabilities provides a fitting case study of the ethic of autonomy because, as he phrases it, they are persons “whose autonomy is always in question” (2010, xii): that is to say, they are individuals who (like all of us) are principally free, yet who are considered to be incapable of exercising their freedom and living independently as a result of their disabilities. Hence, they are deemed to need assistance in becoming autonomous. As a result, argues Levinson, “becoming independent” becomes a life project for persons with intellectual disabilities – and this project is central to group home life. He writes: “In this sense, the group home itself is a technology, a setting organized by the clinical and administrative efforts that govern residents by cultivating the particular capacities that allow each resident to govern him- or herself” (2010, 45).

Levinson observes the ethic of autonomy at work as the central imperative of group home life in everyday practices undertaken by residents and the assistants who care for them, all of which are aimed at honing one’s individual autonomy. He traces it, for instance, in the way residents are taught to be polite and to act responsibly – not through “direct disciplinary prohibitions” but by orienting residents to their own conduct, not dissimilar to how children are socialized by their parents (2005, 69). Such practices in the group home signal a move away from discipline through coercion (considered typical of life in institutions) towards governing through management of the self. For Levinson, group home life thus conceived produces a “tension between freedom and authority” that resembles Rose’s paradox we described above. Residents are governed by making an appeal to their potential for independence – a goal always striven towards but never to be truly reached. All the while, Levinson contends (and here he stands with other authors who discuss intellectual disability care in terms of governmentality), these residents have become newly subjected to a slew of techniques of governance that introduce new manners of constraint and paternalism into their lives. Thus, writes Levinson, for residents of the group home, “freedom is the ability to *participate in their own supervision* through their own specific pursuits of freedom” (2010, 46, emphasis ours). In a similar vein, Drinkwater argues “supported-living arrangements exemplify not an emancipation, nor even a humanitarian reform, as much as a new dispersal of power relations” (2015, 229); while Altermark contends that “the lives of people with intellectual disabilities seem to be embedded in the productive technologies of government”, which “certainly is far from the idea of a withdrawal of the state from the life of the individual” (2017, 1325). While not all of these authors draw directly on Rose’s ethic of autonomy, his work is implicit in their conclusions.

In this reading, there is something tragic about the group home’s attachment to the ideal of “independence”. It offers only a mirage of the good life, never quite realized, which in fact exposes those who pursue it to insidious exercises of power (Levinson 2010, 52). Lauren Berlant developed a concept to talk about such attachments: *cruel optimism*, which she defines as a relation in which “something you desire is actually an obstacle to your flourishing” (2011, 1). Such attachments are cruel because “the object/scene that ignites a sense of possibility actually makes it impossible to attain the expansive transformation for which a person… risks striving”; and “doubly cruel” when such an attachment has become fulfilling to this person in spite of its adverse effects on that person’s life (2011, 2). Cruel optimism thus designates investments into desires, projects, and dreams that are in fact detrimental to one’s well-being. If the ideal of independence, as Levinson has it, is indeed “an endless clinical pursuit toward an ever-receding goal” (2010, 52) – a pursuit that introduces an updated version of disciplinary paternalism to boot – the group home’s adherence to the ethic of autonomy is best understood as a form of cruel optimism. In this reading, “becoming independent” emerges as a “good-life fantasy” (2011, 1) that holds an empty promise. As it turns out, holding on to the ethic of autonomy is actually detrimental to the flourishing of the residents of the group home. This is what we call the cruel optimism thesis of the implications of the ethic of autonomy for the group home.

## The case of Jos and Valerie

How helpful is the cruel optimism thesis for elucidating care practices in the group home? To answer this question, we turn to ethnographic fieldwork conducted by the first and second author in group homes for people with intellectual disabilities in the Netherlands. The fieldwork was part of a government-funded research project on ‘experiences of client dependency’ amongst people with intellectual disabilities, which was carried out in 2017 and 2018 in 12 group homes administered by seven care providers. In total, we shadowed 13 support staff members for one or two days and 28 residents for one or two days. Shadowing involves following participants as they go about their daily business and asking them for their perspectives on the events as they unfold in practice (McDonald 2005). In this way, shadowing results in thick descriptions of both the events themselves and what they mean to those involved (Quinlan 2008; McDonald and Simpson 2014). In the case of staff members, these events included everyday occurrences like care and support routines, debriefing sessions, and doing paperwork; in the case of residents, they included such occurrences as job visits, commuting, and visits to the day centre.

For the present analysis, we will zoom in on a specific case, by discussing a set of situations from our fieldwork that prominently feature two persons: Jos, a resident of group home The Home, and Valerie, a care assistant employed by The Home. By choosing to focus on situations featuring Jos and Valerie, we follow the logic of ‘exemplar methodology’, which, according to Timmerman et al. (2019, 579), insists on studying the ‘construct of interest in its most consistent and intense form’ (see also Bronk 2012). In our case, this means we choose to discuss a sequence of events that stood out from our data because it was highly saturated with talk about “independence”, inviting an analysis in terms of the ethic of autonomy. (In fact, at the onset of our research, one support worker remarked that Jos had been eager to contribute to our research, as “independence” was an important theme for him.) For this reason, the case can illuminate how the ethic of autonomy is given shape in practice. In one sense, we use this sequence of events as what Bent Flyvbjerg (2006, 230) calls a ‘critical case’ and what Harry Eckstein (1975) calls a ‘plausibility probe’ case: a case that allows us to ascertain the explanatory power of a hypothesis by providing a particularly salient example, which the hypothesis is expected to explain. The logic of the critical case maintains that if a hypothesis (such as the cruel optimism thesis) is not valid for this case, then this hypothesis is unsatisfactory. Our main goal, however, is not to draw generalizable empirical conclusions about the ethic of autonomy on the basis of our case. Rather, we aim to theorize it through an instance in which it is strongly manifested and so provide an alternative to the cruel optimism thesis. In this sense, our use of the case is also what Eckstein (1975) calls ‘heuristic’, as we use it for theory building. The case of Jos and Valerie is especially suited for our aims, because it offers multiple perspectives on the same sequence of events. During our research, we shadowed both Jos *and* Valerie on different days. For this reason, we were able to observe how talk of “independence” played out for each of them in concrete situations and also to hear their ideas about the significance of the word “independence” for their respective lives and/or practices. We choose to direct our gaze towards these moments between Jos and Valerie because we saw them from both of their perspectives, at different times. We should note, however, that while we approach our case as ‘critical’, these observations are in no way irregular for our dataset as a whole. In this sense, the arguments presented here also bear on the rest of our observations – such as the scenes that opened this paper.

Before we dive into our observations, some more words on the setting in which we made them. The Home is a group home located in the south of the Netherlands, in a small municipality bordering a larger city. At the time of our fieldwork, the Home had ten residents – five men and five women. All residents were young adults in their late twenties or early thirties. All were thought to have “mild” intellectual disabilities. Each resident lived in his or her own apartment, featuring a kitchen, living room, bathroom, and separate bedroom. The Home was founded by the parents of its ten residents as a so-called “parent initiative”, making use of client-linked budgets distributed by the Dutch government to pay for property and care expenses. The Home was situated in a separate wing of an apartment building otherwise inhabited by elderly persons. The wing consisted of a large communal living space and kitchen on the ground floor and the ten apartments, which were dispersed over the second and third floors of the wing. The communal space also featured a tiny office used by staff members to do paperwork and make private phone calls. The Home provided support from 7:00 until 23:00, with one or two staff members present depending on the time of day. There was no night shift; residents were deemed capable of doing without support in the night.

## Tracing the ethic of autonomy

Valerie is a support worker in her thirties, who had been a staff member at The Home since its inception – over 8 years at the time of the research. At The Home, Valerie had the function of “personal mentor”, meaning that in addition to everyday activities of assistance, she was also tasked with managing and overseeing the ‘personal plan’ for several residents, Jos amongst them. In practice, this meant she was involved with many aspects of Jos’ life, keeping contact with his family, his workplace, his doctor, and so on. In this way, Jos had a more intense relationship to Valerie than to some of the other care assistants, who generally only occupied themselves with assisting him in his day-to-day life.

### Situation 1: debriefing

Valerie’s shift begins at 15:30, with a quick debriefing session with her colleague Elise. In these sessions, which usually take place in between shifts, care assistants update each other on the events of the day, particularly things that are out of the ordinary for The Home. During this session with Valerie and Elise, their discussion centered on (what they perceived as) a disturbance that had taken place the previous night.

*Jos and his neighbor Gijs had been up late, playing games at Gijs’ apartment – contrary to the house rule. Jos and Gijs were allowed to hang out in their apartment together only one night a week, until 10:30 PM, at which point they had to check in with the evening staff and go to bed in their own respective apartments. Last night, however, they had not come downstairs to the common room at 10:30 PM. The morning after, both called in sick to work, and Elise noticed both seem very tired.*

*Valerie did the night shift yesterday. She tells Elise she did not check on Jos and Gijs after they had failed to check in with her at 10:30 PM. She refused to go upstairs “like a police officer”, she explains. Jos wants to be more “independent”, so she feels she should not act as a cop. “Do you want to show that you can be independent? Then you gotta show you can come downstairs independently”, she says.*

*Valerie and Elise discuss possible repercussions for Gijs and, in particular, for Jos. They contemplate whether or not to hand Jos a “yellow card.” The Home has a system of yellow and red cards designed specifically for Jos, in consultation with his parents – according to his parents, he needs clear directives in order to function well. A yellow card counts as a warning. Two yellow cards in a single week lead to a red card. “Then something less pleasant follows”, says Valerie – for instance, exclusion from an activity. Valerie hesitates to call it a punishment: “I don’t like talking in terms of punishment…” But the yellow card is a way to show Jos his behavior has consequences, she explains. She feels “like a bad person” when she hands Jos a yellow card, but she also thinks “he is an adult person and has to make his own choices”. When I ask her why she hesitates to call the yellow card a punishment, she says: “It sounds so Medieval to me. Punishment is an ugly word. So childish… But without consequences, things won’t work, either. Then nobody does their best.” Elise concurs: “You want to stimulate independence, but it’s no use like this.”*

The debriefing session brings to the fore several of the qualities of the group home as a unit of social organization that have been of interest to scholars inspired by Foucault: there are disciplinary house rules (the card system), there are techniques of surveillance (debriefing sessions, diaries), and there is a clearly demarcated hierarchy (Drinkwater 2015). For the present discussion, however, what stands out here is the prevalence of “independence” as a *leitmotif* in Valerie and Elise’s conversation.

The idea of “independence” has two main functions in their conversation. First, “independence” emerges a desire, which Valerie ascribes to Jos, whom she notes wants to be “more independent”. As such, it also becomes a benchmark, a vantage point from which to judge Jos’ actions and enact interventions where appropriate, in order to support him in this desire to become “more independent”. This function of “independence” thus also ushers in the second: “independence” emerges as a support goal, evident from Elise’s remark that “you want to stimulate independence”. In this sense, “independence” is also the benchmark by which the support workers judge their own actions. Jos’ desire for independence and the staff’s support goal thus overlap. This, in Rose’s vocabulary, might be the ethic of autonomy at work. And here arises the central problem for Valerie: she wants to intervene in order to stimulate Jos’ independence, but this very goal also prevents her from acting “as a cop” and administering punishment. Punishment sounds “Medieval” and “childish” to her – old-fashioned, backwards, and demeaning. The desire she thus ascribes to Jos (being “more independent”) and the goal she sets herself thus also trouble her scope for action as a support worker.

### Situation 2: conversation

To see how Valerie’s problem of “independence” finds resolve for her (and Jos), we turn to a second situation, which occurred several hours from the first, on the same day. Jos has by now received a yellow card from another support worker.^3^ In the evening, Valerie heads to Jos’ apartment to discuss the yellow card with him – being his “personal mentor”, she looks to talk to him frequently about his experiences and feelings.

*The three of us are seated at Jos’ kitchen table. Jos says he is about to watch soccer in the restaurant downstairs. Valerie wonders if this is a smart idea, considering Jos was sick today. Jos feels fit, he claims. Valerie asks until what time Jos was with Gijs last night. Until 10:30 PM, says Jos. Valerie wants Jos to check in with the assistants at 10:30. Yesterday things did not go as they should have. That is why Jos got his yellow card yesterday. Valerie tells him she wants him to try to do his best and she wants him to go to work tomorrow.*

*Then Valerie asks Jos what he thinks about his yellow card. Jos tells her he thought it was not fair, because he followed the rules but forgot to check in with her. “You want to be independent, and then you do something like that”, says Valerie. “Yeah,” answers Jos. “That’s not very independent, is it?”, says Valerie. “No.” “When you’re independent, you’re responsible for your agreements.” “Yes.” Valerie is insistent; Jos sounds defeated.*

If the previous observations indicated how the ethic of autonomy steers the rationale of support staff, this conversation demonstrates how it operates in their practical interventions. Valerie attempts to compel Jos to model his actions after an ideal of “independence” she has him subscribe to, first by asserting him that “you want to be independent,” and then by asking him rhetorically: “that’s not very independent, is it?” Valerie reproaches Jos in the name of independence. Her forceful interrogation indicates that a focus on independence does not preclude compulsion or even coercion, although they take more subtle forms. Rather than outright coercion, “independence” appears to become a self-imposed imperative for Jos: his desire to be “more independent” means he has to alter and adapt his behavior to be in line with the demand of “independence”.^4^ If he begrudgingly agrees with Valerie that he has failed to show himself to be “very independent”, it is because he, like Valerie, is committed to some version of the ideal of independence. In this sense, it seems both Valerie and Jos subscribe to the ethic of autonomy Rose and Levinson describe.

## Two concepts of “independence”

So far, we have looked at the notion of independence as a technique of governance. We were interested in how independence figures in the practical rationale of support staff, and how it exercises power over the residents who aspire to it. Mattingly (2014, 47) would call such an analytical vantage point on ethical life a “third-person” perspective: we looked at Valerie and Jos as if from a distance, as representatives of their social categories, behaving according to dominant norms. Such a “third-person” position, Mattingly explains, locates ethical subjectivity in systems, social practices, and discourses – in “powerful preexisting moral codes and practices” (2014, 47). What we have not done is ask what independence *means* to the participants in these scenes, by taking their intentions and their commitments as a starting point. Mattingly (2014) would call this a “first-person” position. Such a first-person position foregrounds moral striving, commitment, and “processes of ethical judgment grounded in singular events” as sources of ethical subjectivity (2014, 55). These subjects emerge in particular histories and traditions, but they do so with some “agentive resilience”: they reinterpret and act on the traditions they have inherited (2014, 55–7). If Valerie and Jos are committed to the idea of independence (as indeed they seem to be) it is well worth wondering what it means to them and why they choose to pursue it in their practices. If these are guided (or indeed governed) by an ethic of autonomy, it could be instructive to look closely at how the ethic of autonomy is interpreted and enacted by them as they navigate their care relationship.

To understand what independence means to Valerie, we can turn to scenes we described above. In their debriefing session, Valerie remarked to Elise (about Jos): “do you want to show that you can be independent? Then you gotta show you can come downstairs independently.” In Valerie’s mind, then, “independence” appears to consist in abiding to rules and agreements and taking responsibility for one’s actions. For her, becoming independent means adopting a sense of responsibility and obligation: it is about carrying the (occasionally burdensome) duties of adulthood (Munson 2020). In other words, for Valerie, independence is tantamount to *self-reliance*. This is why Jos’ conduct creates such a dilemma for her: she wants to promote virtues like responsibility and duty, but she seems to think that enforcing such qualities through punishment would also empty them of their meaning. This concept of independence thus keeps her from acting, as she puts it, “like a cop”. It is as Elise put it: “You want to stimulate independence, but it’s no use like this.”

To understand what independence means to Jos, we turn to our interactions with him, which occurred some weeks later, as we followed him on a regular workday in the supermarket where he is employed. The following situations all occurred on that day; we group them together because the conversation interlock thematically.

### Situation 3: walking to work

*Jos tells me he still has childhood diaries, in which he wrote about his dream future: his own house, a garden, living with a girlfriend. But his life looks different from what he imagined, he says, “with an apartment like that.” He would have preferred a house, one with stairs to get to the bedroom. He wants to do what he feels like doing.*

### Situation 4: lunch break

*Jos tells me he really wants to be in a relationship, “to get married and everything. Then I’ll be rid of the fuss of The Home.” I ask him what he means by “fuss”. “Assistance. That you… that they’re on your case all the time, making phone calls for you.” When Jos was still living with his parents, they asked him whether he would like to live independently. He told them that he did. But he now believes he had been too quick to say yes: he hadn’t realized they meant a place like The Home. “I wanted a house, with a garden and everything. [Support worker] Elise tells me, ‘you can’t, you need assistance.’” I ask Jos if he agrees. “Not entirely,” he says. It’s nice to have your own home and garden, and a girlfriend. That you can decide for yourself when you cook. That you can decide for yourself.*

### Situation 5: walking home

*I ask Jos what he thinks of his personal mentor Valerie. “Valerie is not necessary for me.” I ask him what Valerie does for him. “Help with stuff,” he says. It’s not wrong what she does, he explains. But he could do without the conversations. I ask him what they are about. Activities, weekends, Fridays, he says. About him spending too much time hanging out in cafes. I ask him why. He tells me his mother says so. It’s about “money and stuff.” Sometimes Jos spends too much in cafes, and he runs out.*

Like Valerie, Jos is attracted to the idea of “independence”, and he seems disillusioned by a life that does not fully accommodate it. (It seems the support worker was right in remarking that “independence” was an important theme for him – it even featured in his childhood diaries.) But Jos’s conception of “independence” differs from Valerie’s. For Jos, “independence” is about having the proverbial white picket fence – a house, a family, a garden. An “independent” life is therefore about normalcy and ordinariness, a life like every other. At the same time, though, “independence” for Jos is also about “doing what you feel like doing” and “deciding for yourself”. It is thus about *self-determination* – even if that means living a normal (some might say average) life.

Valerie’s and Jos’s respective conceptions of independence differ in two important ways. The first is a difference in *values*: Valerie’s notion of independence centers on self-reliance (which emphasizes responsibility), while Jos’s centers on self-determination (which emphasizes freedom). This also leads to a second difference, which is *temporal*. For Valerie, “independent” is something you *become*, by acting responsibly and mature – by going to bed on time, going to work, and keeping your promises. “Independence”, in other words, is a project; here, her conception resembles the ethic of autonomy described by Rose, as “independence” engages residents like Jos in a project of self-improvement in a route towards autonomy. For Jos, however, “independent” is something you *are*, by virtue of living a normal life – by virtue of having a house, a girlfriend, and a garden, or by virtue doing what you feel like doing. In a sense, this is the end goal of Valerie’s project. But Jos is not interested in this project, as for him, “independence” does not need a route that involves (what he perceives as) patronizing support workers making a “fuss”. He just needs the house. It is not that Jos does not appreciate the fact that he may need support – he says what Valerie does is not “wrong” – but for him, receiving help and independence are not antithetical. Independence is a state, a way of life, separate from the support that Valerie insists is needed to get there. And for this reason, Valerie’s attempts to engage Jos in a project of “independence” are rather unsuccessful: because Jos understands “independence” differently, he does not see the point. Certainly, both seem, in their own ways, committed to an ethic of autonomy; yet what are the implications of their different commitments?

## The cruel optimism thesis

One way to answer this question would be to invoke what we have called the cruel optimism thesis. As mentioned above, a number of scholars is suspicious of the prevalence of the ethic of autonomy in the group home. They insist that its ideal of independence subjects its residents to new forms of supervision and coercion, by hailing them to modify and adapt their own conduct to realize the freedom to which they aspire. In this way, investing in the idea of independence becomes a form of cruel optimism: a relation in which “something you desire is actually an obstacle to your flourishing” (Berlant 2011, 1). In the present analysis, that seems to hold true both for Jos and for Valerie. By pursuing an ideal of “independence” that involves a vision of normalcy and full self-determination, Jos is devoted to what Berlant would call a “good life fantasy” that seems unattainable in practice (2011, 1). Valerie is intent on supporting Jos to get on the route to “independence”, but since her version of “independence” is of no interest to Jos (and even contrary to his), her attempts seem mostly patronizing and demoralizing from his perspective. This makes their joint attachment to the ethic of autonomy cruel for both. For Jos, “independence” provides an image of a dream future that in practice will remain elusive – yet it subjects him to a project of self-improvement that will not get him much closer to this dream. As for Valerie: her endeavours to coach Jos into independence are received with indifference or even resistance, as he does not share the goal she espouses. She ends up having to act as the “cop” she is reluctant to be. In this way, their individual projects of independence (Jos to achieve it, Valerie to support it) are bound to fail, but so is their joint one. As their concepts of independence are incompatible, their care relationship becomes fraught with tensions, and sticking with it will achieve the opposite of what they desire – cruel optimism, indeed.

Our observations do appear to lend some credence to such a position. Yet we also think it glances over an important aspect of how the ethic of autonomy is enacted in practice. The cruel optimism thesis requires Valerie’s and Jos’s particular conceptions of independence to be *static*: their strivings can only be futile if their separate goals remain unchanged, the values that drive them – self-reliance and self-determination, respectively – unyielding. Such an image of group home practice is not entirely adequate, though. To show why, we turn to one final scene of our observations of Valerie and Jos.

### Situation 6: dinnertime

*During dinner, Jos gets a phone call from his sister, who’s asking him for help with some jobs around the house the next day. Jos is eager to help, and they make arrangements. After he hangs up, he tells Valerie about it. She asks Jos what time he needs to get up to catch the bus, and whether he has old clothes to wear the next day. Jos responds by suggesting he could also travel to his sister after dinner, so he doesn’t have to get up early the next day. Valerie says he could, but he’d have to call her first, and she tells him to check the bus schedule, too. Jos thinks it won’t matter to his sister. She told him he could come any time. He doesn’t call her. After Valerie urges him some more, Jos opens a phone application to check the bus schedule. Valerie shows him which routes have the least changes and will be easiest for him. Together they pick the most favorable one.*

It is possible to interpret this scene in terms of the cruel optimism thesis. Such an interpretation would notice how Valerie exercises power over Jos in the name of self-reliance – encouraging responsible behavior, navigating his social obligations. Such an analysis might point at how she intervenes and imposes restrictions and at how she attempts to shape his conduct in specific ways. It may even discern Jos’s attempts to resist these attempts, by disregarding her advice and making his own plan. In such a reading, Valerie and Jos are engaged in a moral conflict of sorts, fueled by their discordant notions of independence.

However, we wish to propose an alternative: one that reads the scene as a process of mutual adaptation. Such a reading does not see conflict, but compromise, and perhaps even a process of transformation. It notices how Valerie and Jos both keep on adapting their goals and reattune their values in an attempt to accommodate the other’s. So Valerie accepts that Jos leaves tonight, and that he will not call his sister to confirm his change of plans; Jos, meanwhile, resigns to the help Valerie offers him in figuring out his transportation, allowing her to organize his life in ways she considers fitting. Both Jos and Valerie appear to settle for something not quite like the value that underpins their concept of independence – self-determination for Jos, self-reliance for Valerie. Borrowing a concept from Mattingly (2013; 2014) we call this interpretation the moral laboratory thesis.

## The moral laboratory thesis

Mattingly introduces the moral laboratory as a metaphor for ethical practice. “Ethical practice” here must be understood in a broad sense, referring to the moral dimension of everyday acts and behaviors.^5^ Central to this metaphor is the experimental nature of such practices. Another core tenet is that it views ethical practice as bound up with attempts at living a good life. By referring to social spaces as moral laboratories, she wishes to portray them as “spaces of possibility, ones that create experiences that are also experiments in how life might or should be lived” (2014, 15). In these spaces, actors emerge as “researchers or experimenters of their own lives” (2014, 16), engaging in everyday forms of moral experimentation in their attempts to give shape to a life they consider good. (Mol, Moser & Pols (2010) might call this “tinkering”.) It is thus an imaginary of moral life that locates “ethics” in mundane, everyday practices through which people try to live well (or at least better), by taking a leap into the unknown, taking a risk, or perhaps simply deciding to do *something*. As Michael Lambek (2017) puts it, “when the path forward is unclear, sometimes you just throw something out there, a kind of trial balloon, to see how it works, how others respond, and how you do too.” As a site of moral reflection and moral practice, the moral laboratory holds the promise of transformation, of the emergence of something new – drawing on Hannah Arendt (1998), she speaks of the “miracle of natality” (2014, 16).

Mattingly (2013) contrasts the moral laboratory with two different metaphors for ethical practice she discerns in moral anthropology: the “trial” and the “artisanal workshop”. In the trial (which she connects to Nietzsche), moral subjects emerge by being made to recall and defend their own actions in the face of an accusation and possible punishment; in the artisanal workshop (which she connects to Foucault), moral subjects make *themselves*, as they hone their conduct and their bodies according to specific rules and norms in order to cultivate a virtuous character. In both of these metaphors, “power is the main protagonist” (Kuan and Grøn 2017, 189): moral subjects emerge in relation to power, be it an accusing figure of authority or a set of norms and rules. For Mattingly, however, neither of these metaphors capture “the vagaries of everyday life and the difficulties of discerning what might constitute the most morally appropriate action in the singular circumstances life presents” (Mattingly 2013, 304) She presents the moral laboratory both to underline the *everyday* nature of ethical practice and its *transformative* potential, neither of which she believes are adequately evoked in the other metaphors.

If we apply Mattingly’s typology to the cruel optimism thesis, we can see that it relies on a vision of moral life that resembles the artisanal workshop. It describes people who engage in a project of self-fashioning, adopting a set of relatively static social norms by which they shape their behavior and form their character. Such a perspective highlights the imbrication of ethical life with power structures that transcend (or even override) individual intentions and commitments. This is how Jos comes to be gripped with the ethic of autonomy, which subjects him to subtle forms of compulsion and coercion. For authors like Levinson, this is what the ethic of autonomy attempts to describe: a set of norms about the subject’s relation to freedom, which drives him or her to particular forms of conduct. It is also why they are suspicious of the prevalence of the ethic of autonomy in the group home. But if this set of norms constitutes an *ethic*, such authors have surprisingly little to say about the ethical practices in which these norms express themselves; how they are interpreted and enacted by people in their striving to bring about something good. In other words, while this metaphor of ethical life provides a useful lens on the workings of power in the shaping of ethical conduct, it has problems bringing Valerie and Jos into view as actors who try to improve on their lives by pursuing a particular conception of the good. In Mattingly’s vocabulary, the cruel optimism thesis seems to approach them mostly as “third-person” actors, who are shaped (or indeed governed) by their social conditions. By disregarding everyday moral practice, this thesis “misses the many ways people experiment with, critique, and modify the very traditions they have inherited or in which they have “schooled” themselves as part of their self-making projects” (Mattingly 2014, 79).

Approaching the group home as a moral laboratory, however, gives us a clearer look at Valerie and Jos as “first-person” moral actors who act upon their conditions. The moral laboratory metaphor thus opens up a different perspective on Valerie’s and Jos’ preoccupation with “independence”. Looking at the scenes we narrated, we can begin to see how their conversations have moral import to Valerie and Jos. This is clear, for instance, from Valerie’s self-doubt when considering how best to approach the ‘situation’ with Jos; and also from Jos’ thoughts on the support he receives from Valerie, which he describes in moral terms as “not wrong”. We see, in other words, two actors engaged in an attempt to pursue what they consider to be “good”, both for themselves and for the other. For Valerie, this good is “independence” as self-reliance, which she encourages in her support interventions with the aim of altering Jos’ behavior; for Jos, this good is “independence” as self-determination, which he pursues through his resistance and defiance against Valerie’s attempts to produce self-reliance. But in scenes like these, Valerie and Jos are not merely reproducing a social tendency (the ethic of autonomy) in their practices: they are shaping and reshaping that ethic as they live their lives and strive to make these lives better. They may disagree about what makes for a better life; what matters is that they participate in a collaborative experiment to shape it. Such a reading, in other words, depicts Valerie and Jos not as being subjected to, but as experimenting with the ethic of autonomy, exploring new ways of how it might shape and alter their lives, in the pursuit of living well.^6^

However, the moral laboratory does not necessarily constitute an equal playing field. Hence, to take on such a “first-person” perspective on group home practice is not to erase the problem of power. Hierarchy matters in the group home – that much became clear from the first two scenes between Valerie and Jos, in which Valerie fiercely reproached Jos for not measuring up to *her* standard of independence. At the end of the day, as a result of their respective social roles, Jos (almost literally) has to answer to Valerie when it comes to living his daily life. The outcomes of their moral experiments with the ethic of autonomy are conditioned by this hierarchy, too. This gives Valerie a lot more leverage to steer their joint experiments, as well as to control their outcomes. For this reason, the results of their experiments can still be tragic. Yet they are not *inherently* so. It is precisely the unpredictability and openness of moral experimentation that challenges the cruel optimism thesis. Taking a “first-person” view on exchanges in the group home allows us to take these actors’ commitment to the idea of independence seriously and to recognize how it shapes their efforts to live a life they consider worth living. While these efforts *can* be marred by inequality, they need not be; the final scene between Jos and Valerie gives us a glimpse at that.

In this way, the metaphor of the moral laboratory lets us depict the group home as a place where different conceptions of “independence” (such as independence as self-reliance and independence as self-determination) are proposed, contested, and negotiated in practices of care and support – although the capacity for doing so is not distributed equally amongst social actors. This process of adaptation expresses itself in small-scale, minute experiments that have the power transform the ordinary, for better or for worse. One such experiment could be to disregard your support worker’s advice – or to take it. The transformative aspect of the moral laboratory lies in such creative re-imaginings of what “independence” might look like or how it could be practiced. Such a reading depicts Valerie and Jos not merely as being subject to an ethic of autonomy that “conducts their conduct”, but as experimenting with the ethic of autonomy in the pursuit of living well.

Our take on the ethic of autonomy in terms of moral experimentation should not be understood as an attempt to formulate a normative judgment about the pursuit of “independence” in the group home as such. Mattingly devised the moral laboratory as a metaphor for moral life. While this metaphor can shed life on critical practices in the everyday (Mattingly 2014, 207), we use it principally as a *heuristic* device. Our point is not to evaluate moral practice, but to bring moral practice into view in a different way – and in so doing, to show the ambiguity and open-endedness of the pursuit of the ideal of independence. If our attempt to cast the group home as a moral laboratory (and to approach Jos and Valerie from a “first-person” perspective) contains a normative dimension, it resides in our conviction that we ought to pay attention to this “first-person” view, by taking the hopes and strivings of moral actors at face value. Doing so reveals the weaknesses of the cruel optimism thesis and its governmentality frame: first, that its portrayal of the ideal of “independence” is too monolithic and second, that its account of moral practice is overdetermined in terms of power. None of this implies a definitive judgment about the ethic of autonomy in the group home itself. Moral experimentation with the ideal of “independence” is fundamentally open-ended, which means its outcomes can certainly be tragic, too. So when we disagree with the cruel optimism thesis, we do so on account of its depiction of moral life; not on account of its skepticism towards the ideal of “independence” as such.

## Conclusion: governmentality in care practices

The cruel optimism thesis is a tempting one, because it gleefully shatters the narrative of indisputable progress that tends to surround the move from the institution to “the community” (Altermark 2017). It is critical of the language of self-determination and autonomy that characterizes contemporary intellectual disability care, which it considers to be inaccurate or, at worst, deceptive. This reading provides a strong perspective from which to scrutinize contemporary policies and practices in intellectual disability care, as has been done by writers like Dowse (2009), Yates (2015), and Levinson himself. From our observations, too, there is reason to assume that this criticism has some plausibility. Yet as we have shown, this position also has its flaws. It risks failing to account hermeneutically for what the ethic of autonomy means to actors, as well as what drives them to commit to it and actualize it. If we do take such a “first-person” view on moral practice, we can see that the ethic of autonomy informs a set of practices around a contested “good” (independence), which has multiple interpretations, and constantly gets renegotiated in practice.

The metaphor of the moral laboratory also casts the scenes that opened this paper in a different light. We noted there that there was something curious, even ironic, about the contradictions of independence they each contained. In one sense, we could view these scenes as moments that ridicule these support workers’ preoccupation with the ethic of autonomy and where the promise of “independence” falls flat – the cruel optimism thesis. In another, though, these scenes provide small windows on the group home as a moral laboratory, where support workers and residents alike are experimenting with ways in which the shared “good” of independent living might be put into practice. For instance, by using your own plate and cutlery, even if the food is prepared by a support worker. Such laboratories are transformative when they produce practices that reimagine “independence” in ways that work for their participants. How Dennis and his support worker Eric navigate the issue of bathing might be one such creative solution; Jessica’s clumsy attempt to respect Erdem’s input indicate that these experiments may not always work out. In such a reading, the tensions and ironies apparent in these scenes become symptomatic for a social space in which people are trying their best to explore in practice “how life might or should be lived” (Mattingly 2014, 5). Seen in this way, these scenes depict support workers and residents experimenting with the ethic of autonomy to find creative solutions to a fundamental condition of group home life: that the need for support necessarily implies some form of dependency.

This moral laboratory thesis might be provocative to some. Since it shifts emphasis from power and structure to intention and personal moral striving, there could be a danger of glancing over power imbalances that lead to the forms of coercion, supervision, and domination reported by authors like Levinson (2010), Drinkwater (2015), and Altermark (2017). Such authors might retort that adopting the ethic of autonomy is a matter of power, rather than intention. Yet looking at intellectual disability care only through the lens of power makes it difficult to bring into view the everyday struggles both of support workers and of group home residents in giving shape to a life they consider worth living. In other words, it is in danger of failing to take “people’s moral projects and their beliefs about the good seriously” (Mattingly 2014, xvi). When we do, it becomes apparent that the attachment to the idea of “independence” is not mere cruel optimism – even if the results might no doubt still be tragic, as they perhaps were when Jessica put Erdem’s suggestions aside, or when Valerie challenged Jos: “that’s not very independent, is it?”.

This also brings us to a broader point about the use of the governmentality vocabulary for analyzing care practices. To say that the cruel optimism thesis misses an important dimension of the moral practices that characterize the group home is not to say that governmentality as such is not a helpful conceptual frame. To the contrary: it was quite evident in our observations that the ethic of autonomy holds sway in the group home. However, since it does not appear to offer a hermeneutics of ethical commitment and intention, there is a limit to what a “third-person” perspective such as the governmentality frame can illuminate about ethical practice. “Third person or discursive positions… favor systems, social practices, or the technologies within particular “regimes of truth” (to borrow Foucault’s language) as the primary agentive sites”, Mattingly writes (2014, 47). Such accounts of ethical subjectivity have limited interest (or outright disregard) for intention and inner life.^7^ Since so much of caring has to do with emotion, intuition, and attitude, such a “third-person” perspective seems too limited to evaluate them convincingly.

This is not to say that power ought to be disregarded when analysing care practices. To the contrary: power conditions care in fundamental ways, both structurally (in terms of the political distribution of care and care duties) and interpersonally (as every care relation constitutes a relation of power) (Tronto 2013). But if we want to understand why care is practiced in the way it is, how it is experienced, and what people want from it, we cannot rely on a purely “third-person” view on things. As Andrew Sayer (2011) might put it, we need to know why things such as “independence” matter to people. If we take people’s actions seriously as moral strivings towards something good, we can begin to see how much of what happens in the group home (and beyond) is not a mere exercise of power, but an experiment in trying to live life a little better.

## Footnotes

1 All names, including the name of the group home, are pseudonyms. Jos and Valerie were both shadowed by the first author.

2 Foucault’s exact words here are “mechanisms and procedures intended to conduct men, to direct their conduct, to conduct their conduct” (2014: 12).

3 The first author was shadowing Valerie and hence not present to witness this event.

4 Ironically, though, Valerie has not refrained from applying distinctively disciplinary measures – after all, Jos had also been handed a yellow card, albeit by a colleague. This apparent contradiction has been spotted by Levinson, too, who notes that the fact that “government depends on the capacity for freedom does not necessarily preclude the use of constraint or coercion” (2010). What matters is that even if some measures can be explicitly disciplinary in nature, they are aimed at pointing their subjects to their self-conduct in terms of becoming “more independent”. If there is a paradox here, it is not very different from the paradox of freedom inherent to governance as already spelled out by Rose.

5 So in Mattingly’s theory, “ethical practice” emphatically does *not* refer to something like moral reflection or deliberation, in which a normative conclusion must be reached. Instead, it alludes to the moral dimensions of everyday life – a moral anthropology.

6 In such a reading, Jos and Valerie’s mutual striving towards independence comes to resemble what Catriona Mackenzie and Natalie Stoljar (2000) call ‘relational autonomy’, in which autonomy is a function and a product of the quality of the social relations in which agents are embedded. For reflections on relational autonomy and disability, see Davy (2015), Winance (2016), and Munson (2020).

7 It is no coincidence that Mattingly frames her work on the moral laboratory explicitly as a critique on moral anthropologies inspired by Foucault, which, in her mind, fail to consider the importance of intention and inner life for evaluating moral practices. A full examination of her critique goes beyond the scope of this paper, but see Mattingly (2014), especially chapter two.

## References

[CR1] Altermark Niklas (2017). The post-institutional era: visions of history in research on intellectual disability. Disability & Society.

[CR2] Altermark, Niklas. 2018. *Citizenship inclusion and intellectual disability: biopolitics post-institutionalisation*. Routledge Advances in Disability Studies. London ; New York: Routledge, Taylor & Francis Group.

[CR3] Arendt Hannah (1998). The human condition.

[CR4] Berlant Lauren (2011). Cruel optimism.

[CR100] Bredewold, Femmianne, Loes Verplanke, Thomas Kampen, Evelien Tonkens, Jan Willem Duyvendak. 2020. The care receivers perspective: how care-dependent people struggle with accepting help from family members, friends and neighbours. *Health & Social Care in the Community* 28(3): 762–770 10.1111/hsc.12906 PMC7187222 31815344

[CR5] Bronk Kendall (2012). The exemplar methodology: An approach to studying the leading edge of development. Psychology of Well-Being: Theory, Research and Practice.

[CR6] Buntinx Wil H. E, Schalock Robert L (2010). Models of Disability, Quality of Life, and Individualized Supports: Implications for Professional Practice in Intellectual Disability. Journal of Policy and Practice in Intellectual Disabilities.

[CR7] Davy Laura (2015). Philosophical Inclusive Design: Intellectual Disability and the Limits of Individual Autonomy in Moral and Political Theory. Hypatia.

[CR8] Dean, Mitchell. 2010. *Governmentality: power and rule in modern society*. 2nd ed. London ; Thousand Oaks, Calif: SAGE.

[CR9] Dowse Leanne (2009). ‘Some people are never going to be able to do that’. Challenges for people with intellectual disability in the 21st century. Disability & Society.

[CR10] Drinkwater Chris, Tremain Shelley (2015). Supported Living and the Production of Individuals. Foucault and the Government of Disability.

[CR11] Eckstein, Harry. 1975. Case Study and Theory in Political Science. In *Handbook of Political Science*, ed. F. J. Greenstein and N. W. Polsby, 7:79–137. Reading, MA: Addison-Wesley.

[CR12] Flyvbjerg Bent (2006). Five Misunderstandings About Case-Study Research. Qualitative Inquiry.

[CR13] Foucault Michel (1982). The Subject and Power. Critical Inquiry.

[CR14] Foucault, Michel. 2009. *Security, Territory, Population: Lectures at the College De France, 1977–78*. Edited by Arnold I Davidson. London: Palgrave Macmillan UK : Imprint : Palgrave Macmillan.

[CR15] Foucault, Michel. 2010. *The birth of biopolitics: lectures at the Collège de France, 1978–79*. Edited by Michel Senellart. Translated by Graham Burchell. New York, NY: Palgrave Macmillan.

[CR16] Foucault, Michel. 2014. *On The Government of the Living: Lectures at the Collège de France, 1979–1980.* Edited by Arnold I Davidson. Translated by Graham Burchell. Basingstoke: Palgrave Macmillan.

[CR17] Graham Helen (2010). How the tea is made; or, the scoping and scaling of ‘everyday life’ in changing services for ‘people with learning disabilities’. British Journal of Learning Disabilities.

[CR18] Kittay Eva Feder (2011). The Ethics of Care, Dependence, and Disability. Ratio Juris.

[CR19] Kittay Eva Feder (2019). Learning From My Daughter: the Value and Care of Disabled Minds.

[CR20] Kuan Teresa, Grøn Lone (2017). Introduction to “Moral (and Other) Laboratories”. Culture, Medicine, and Psychiatry.

[CR21] Lambek Michael (2017). Comments on Moral (and Other) Laboratories: Culture, Medicine and Psychiatry. Culture, Medicine, and Psychiatry.

[CR22] Lemke Thomas (2002). Foucault, Governmentality, and Critique. Rethinking Marxism.

[CR23] Levinson Jack (2005). The Group Home Workplace and the Work of Know-How. Human Studies.

[CR24] Levinson Jack (2010). Making Life Work: Freedom and Disability in a Community Group Home.

[CR25] Mackenzie, Catriona, and Natalie Stoljar. 2000. Introduction: Autonomy refigured. In *Relational autonomy: Feminist perspectives on automony, agency, and the social self.*, ed. Catriona Mackenzie and Natalie Stoljar, 3–31. New York: Oxford University Press.

[CR26] Mattingly Cheryl (2013). Moral Selves and Moral Scenes: Narrative Experiments in Everyday Life. Ethnos.

[CR27] Mattingly Cheryl (2014). Moral laboratories: family peril and the struggle for a good life.

[CR28] McDonald Seonaidh (2005). Studying actions in context: a qualitative shadowing method for organizational research. Qualitative Research.

[CR29] McDonald Seonaidh, Simpson. (2014). Shadowing research in organizations: the methodological debates. Qualitative Research in Organizations and Management: An International Journal.

[CR30] Meininger Herman Paul (2001). Autonomy and professional responsibility in care for persons with intellectual disabilities. Nursing Philosophy.

[CR31] Meininger Herman Paul (2010). Connecting stories: A narrative approach of social inclusion of persons with intellectual disability. Alter.

[CR32] Mol Annemarie, Moser Ingunn, Pols Jeannette (2010). On Tinkering in Clinics, Homes and Farms.

[CR33] Munson Adrianna Bagnall (2020). Framing Life as Work: Navigating Dependence and Autonomy in Independent Living. Qualitative Sociology.

[CR200] Newman, Janet, and Evelien Tonkens. 2011. (Eds.) Participation, responsibility and choice: summoning the active citizen in western European welfare states. Amsterdam: Amsterdam University Press.

[CR34] Pols Jeannette, Althoff Brigitte, Bransen Els (2017). The Limits of Autonomy: Ideals in Care for People with Learning Disabilities. Medical Anthropology.

[CR35] Quinlan Elizabeth (2008). Conspicuous Invisibility: Shadowing as a Data Collection Strategy. Qualitative Inquiry.

[CR36] Reinders Hans (2002). The good life for citizens with intellectual disability. Journal of Intellectual Disability Research.

[CR37] Rose Nikolas (1999). Powers of Freedom: Reframing Political Thought.

[CR38] Rose Nikolas (2005). Governing the soul: the shaping of the private self.

[CR39] Sayer, R. Andrew. 2011. *Why things matter to people social science, values and ethical life*. Cambridge, UK; New York: Cambridge University Press.

[CR40] Schalock RL (2004). The concept of quality of life: what we know and do not know. Journal of Intellectual Disability Research.

[CR41] Spivakovsky Claire (2017). Governing freedom through risk: Locating the group home in the archipelago of confinement and control. Punishment & Society.

[CR42] Timmerman Guus, Baart Andries, Vosman Frans (2019). In search of good care: the methodology of phenomenological, theory-oriented ‘N=N case studies’ in empirically grounded ethics of care. Medicine, Health Care and Philosophy.

[CR43] Trent, James W. 1994. *Inventing the feeble mind: a history of mental retardation in the United States*. Medicine and Society 6. Berkeley: University of California Press.

[CR44] Tronto Joan (2013). Caring Democracy: Markets, Equality, and Justice.

[CR300] Van der Weele, Simon, Femmianne Bredewold, Carlo Leget, and Evelien Tonkens. 2020. What is the problem of dependency? Dependency work reconsidered. *Nursing Philosophy*. 10.1111/nup.12327. 10.1111/nup.12327 PMC8243965 32935457

[CR45] Winance Myriam (2016). Rethinking disability: Lessons from the past, questions for the future. Contributions and limits of the social model, the sociology of science and technology, and the ethics of care. Alter.

[CR46] Yates Scott, Tremain Shelley (2015). Truth, Power, and Ethics in Care Services for People with Learning Difficulties. Foucault and the Government of Disability.

